# La tuberculose primaire de la glande parotide: à propos de deux cas

**DOI:** 10.11604/pamj.2014.18.237.4829

**Published:** 2014-07-22

**Authors:** Nabil Touiheme, Mounir Kettani, Abdelhamid Messary

**Affiliations:** 1 Service d'Orl et de Chirurgie Cervico-Faciale, Hôpital Militaire Moulay Ismaïl, Meknès, Maroc

**Keywords:** Glande parotide, tuberculose, parotid gland, tuberculosis

## Abstract

La localisation isolée de la tuberculose au niveau de la glande parotide est extrêmement rare. Aucun élément clinique, radiologique et biologique ne permet d'affirmer le diagnostic. Nous rapportons deux cas survenus chez un patient âgé de 40 ans, et une femme de 35 ans immunocompétent, opérés pour une tuméfaction parotidienne et le diagnostic de tuberculose reposait sur l'examen histologique. La symptomatologie de la tuberculose de la glande parotide est polymorphe ce qui pose un problème diagnostic, Le diagnostic est surtout anatomopathologique et le traitement repose sur les antibacillaires.

## Introduction

La localisation isolée de la tuberculose au niveau de la glande parotide est extrêmement rare. Aucun élément clinique, radiologique et biologique ne permet d'affirmer le diagnostic. Nous rapportons deux cas de tuberculose primaire de la glande parotide droite chez un adulte de 40 ans, et une femme de 35 ans, dont le diagnostic était anatomopathologique, on insistant sur les difficultés diagnostic de cette affection.

## Patient et observation

### Observation 1

**Figure 1:**
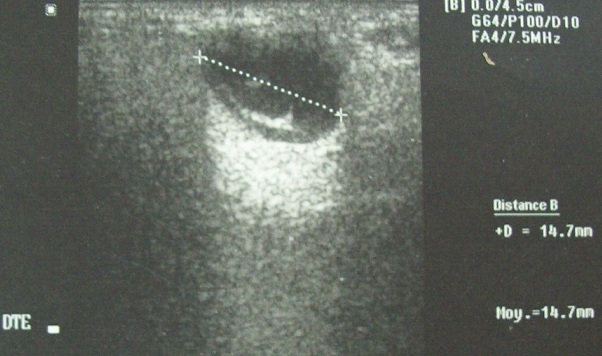
Échographie de la parotide droite: image kystique, avec de fines échos à l'intérieure

**Figure 2:**
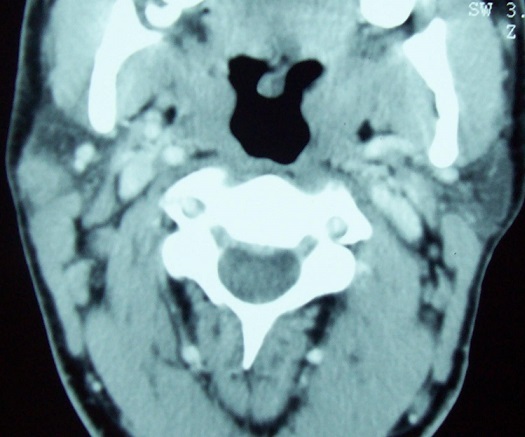
Coupe TDM avec injection du produit de contraste: masse homogène, bien limitée de la parotide droite rehaussée par le produit iodé

Mr I M, âgé de 40 ans, sans antécédent pathologique particuliers, présentait une tuméfaction parotidienne droite évoluant depuis 9 mois dans un contexte d'apyrexie et de conservation de l’état général. L'examen clinique retrouvait une tuméfaction du pôle inférieure de la glande parotide droite, de consistance molle, mesurant 2cm de grand axe, mobile et non douloureuse. A l'examen clinique, on ne notait pas de fistule cutanée ou d'adénopathie cervicale. Le reste de l'examen ORL était sans particularité. L’échographie cervicale retrouvait une glande parotide de taille normale siège d'une image liquidienne avec des échos à l'intérieur, présentant un net renforcement postérieur. Cette masse est à paroi fine et intéresse le pôle inférieur de la glande (Figure 1). Une tomodensitométrie cervicale réalisée avec des coupe de 3 mm d’épaisseur avant et après injection du produit de contraste, retrouvait que la lésion est de densité tissulaire, homogène, bien limitée, rehaussé après injection du produit iodé (Figure 2).

Le reste de la glande était homogène et il n'existait pas d'adénopathie cervicale. La radiographie thoracique réalisée dans le cadre du bilan pré anesthésique était normale. Le bilan biologique standard était sans particularité. Le malade fût opéré, bénéficiant d'une parotidectomie conservatrice du nerf facial. L’étude anatomopathologique concluait à une tuberculose caséo-folliculaire ganglionnaire lymphatique intra parotidienne. Le complément thérapeutique reposait sur une médication antibacillaire RHZ pour une duré de 6mois et l’évolution était favorable.

### Observation 2

**Figure 3:**
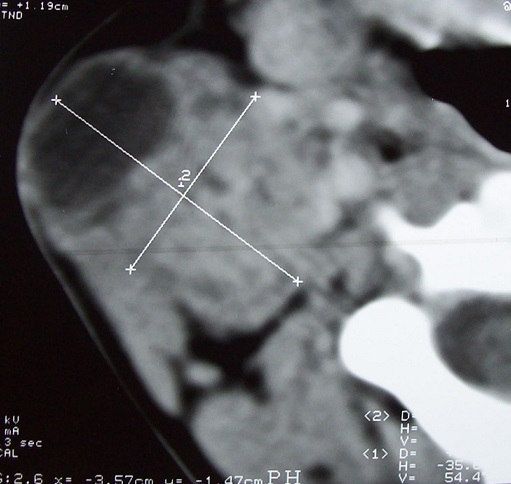
Coupe TDM avec injection du produit de contraste: masse hétérogène de la glande parotide droite

Mme ML, âgée de 35 ans, sans antécédent pathologique particuliers, présentait une tuméfaction parotidienne droite, évoluant depuis une année. A l'examen clinique, la patiente était en bon état général, apyrétique. On notait la présence d'une fistule cutanée en regard de la tuméfaction parotidienne qu’était molles et indolore. L’échographie cervicale montrait que la glande parotide était augmentée de volume siège d'un processus hypoéchogène hétérogène renfermant des zones de nécroses. Le scanner du massif facial notait que le processus mesurait 3,5 cm de grand axe, à double composante tissulaire et liquidienne, déformant le plan cutané en regard, sans adénopathie cervicale associé (Figure 3).

La patiente fût opérée bénéficiant d'une parotidectomie conservatrice du nerf facial. L’étude anatomopathologique de la pièce opératoire concluait à une tuberculose parotidienne et la patiente a été mise sous traitement antibacillaire pendant une durée de 6 mois et l’évolution était favorable.

## Discussion

La tuberculose est une infection granulomateuse chronique du à mycobacterium tuberculosis ou bovis qui peut toucher tous les organes. L'atteinte des glandes salivaires et notamment de la parotide est extrêmement rare. Cette affection est décrite pour la première fois en 1894 par Von Stubenrauch; elle se voit souvent chez le sujet jeune entre 20 et 40 ans et rentre généralement dans le cadre d'une tuberculose disséminée. Dans ce cas, la diffusion se fait par voie hématogène ou lymphatique à partir d'un foyer pulmonaire, rarement elle est primitive comme c'est le cas de notre patient [1, 2].

La présentation clinique est polymorphe et non spécifique; généralement elle se manifeste par une tuméfaction parotidienne unilatérale progressive réalisant un syndrome pseudo tumoral. Les signes d'imprégnation tuberculeuse sont rarement présents et la présence d'une fistule cutanée est très évocatrice d'une pathologie inflammatoire [3].

La tuberculose parotidienne peut être classé en deux formes: une forme focale qui correspond a une infection tuberculeuse d'un nodule lymphatique intra glandulaire et une forme diffuse qui correspond à une atteint de l'ensemble du parenchyme parotidien. Cette dernière forme est rare et semble en rapport avec une dissémination hématogène [4].

Le diagnostic différentiel clinique des formes diffuses inclus: la parotidite infectieuse, lithiasique et le carcinome. La forme circonscrite évoque surtout un kyste ou une adénite [5]. La localisation tuberculeuse au niveau des deux glandes parotides est possible posant un problème diagnostique avec le syndrome de Gougron Sjogren et la sarcoïdose. En générale toute parotidite résistante au traitement antibiotique doit alerter le clinicien vers une tuberculose [5, 6].

Sur le plan biologique on retrouve généralement un syndrome inflammatoire et l'intradermoréaction à la tuberculine n'est pas toujours positive. Les moyens d'imageries en coupe: échographie, TDM et IRM sont peu contributifs au diagnostic positif. En effet les aspects radiologiques sont variables; elle peut s'agir d'un nodule tissulaire, d'une image kystique, plus ou moins associées à des adénopathies intra parotidiennes. Cependant, Bhargava rapporte sur 100 images scannographiques, que la présence d'une lésion à paroi épaisse, prenant fortement le contraste avec de la nécrose au centre, est caractéristique de la tuberculose [7].

La radiographie thoracique doit être systématiquement demandée à la recherche d'un éventuel foyer primitif. La cytoponction parotidienne à l'aiguille fine avec une étude cytologique à la recherche de BAAR et mise en culture du liquide de ponction peut être utile mais n'a de valeur que s'elle est positive, elle a une spécificité de 80% une sensibilité de 94%; en plus elle fait courir au malade un risque de lésion du facial ou de fistulisation [8, 9]. Certains auteurs évoquent la possibilité de confirmation diagnostique par amplification génique (PCR) après culture de broyât cellulaire glandulaire ou de prélèvement de pus au niveau du méat du canal de sténon [10].

Dans certains cas, seul l'examen histologique d'une adénopathie ou parfois même d'une parotidectomie permettra de poser le diagnostic en mettant en évidence le granulome épithélio-giganto-cellulaire avec nécrose caséeuse spécifique de l'atteinte tuberculeuse [10]. Le traitement repose sur les antibacillaires pendant une durée de 6 à 9 mois, permettant la stérilisation du foyer tuberculeux et la disparition rapide du syndrome tumorale parotidien. Ce traitement est réalisé en deux phases: la première phase utilise quatre médicaments (l’éthambutol, l'isoniaside, la rifampicine et le pyrazinamide) pendant deux mois. Durant la deuxième phase, les médicaments utilisés sont l’éthambutol et l'isoniaside pendant sept mois ou bien l'isoniaside et la rifampicine pendant quatre mois [5]. Si l'infection est causée par une mycobactérie atypique, ce traitement antituberculeux est insuffisant et l'exérèse du parenchyme pathologique s'impose [10].

## Conclusion

La tuberculose parotidienne est rare. Elle pose un problème diagnostique car ni la clinique, ni la biologie, ni l'imagerie ne permet d'affirmer l’étiologie tuberculeuse d'un syndrome tumorale parotidien. Le diagnostic est anatomopathologique et le traitement repose sur les antibacillaires.
